# Migrating pseudolipoma of Glisson's capsule: A case report

**DOI:** 10.1016/j.radcr.2024.08.028

**Published:** 2024-08-29

**Authors:** Qiongying chen, Bo zhao, Yao chen, Xing yang, Ke Zhou

**Affiliations:** Department of Radiology, Third People's Hospital of Zigong, Sichuan Province, China

**Keywords:** Pseudolipoma of Glisson's capsule, Migrated, Glisson's capsule, Laennec's capsule

## Abstract

Pseudolipoma of Glisson's capsule is a rare, benign subcapsular liver lesion that typically occurs in older adult men. It comprises degenerated fat tissue that likely originates from detached mesothelial appendages or degenerated liver lipomas. We report the case of a 58-year-old female patient with a gastric malignant tumor after admission. No lesions were found in the liver capsule before surgery. During postoperative reviews from 2015 to 2018, new dense, fatty lesions were found under the liver capsule, and highly unusually, the lesions moved under the liver capsule over time. To the best of our knowledge, only 1 other case has been reported of a pseudolipoma of Glisson's capsule that migrated over time. This supports the hypothesis that migrating mesothelial attachments form Glisson capsule pseudolipomas. This case report aims to review liver capsule anatomy, explain why the liver is particularly susceptible to this phenomenon, and present information to aid the diagnosis of fat-containing hepatic lesions by providing a unique perspective on certain pathologies affecting the liver.

## Introduction

A pseudolipoma of the Glisson's capsule (PGC) is a mass of fat tissue encapsulated and enveloped by the liver [1]. It originates from part of the epiploic appendix that degenerates and later calcifies between the diaphragm and the liver. Other causes may include traumatic inclusion of fat within the liver capsule during surgery, or by transcutaneous liver biopsy [2]. Few cases have been reported; these suggest a prevalence of 0.2% and mean age of 67 years old [3,4]. Treatment is generally not indicated as it is usually asymptomatic [5].

## Case report

**Fig. 1:**
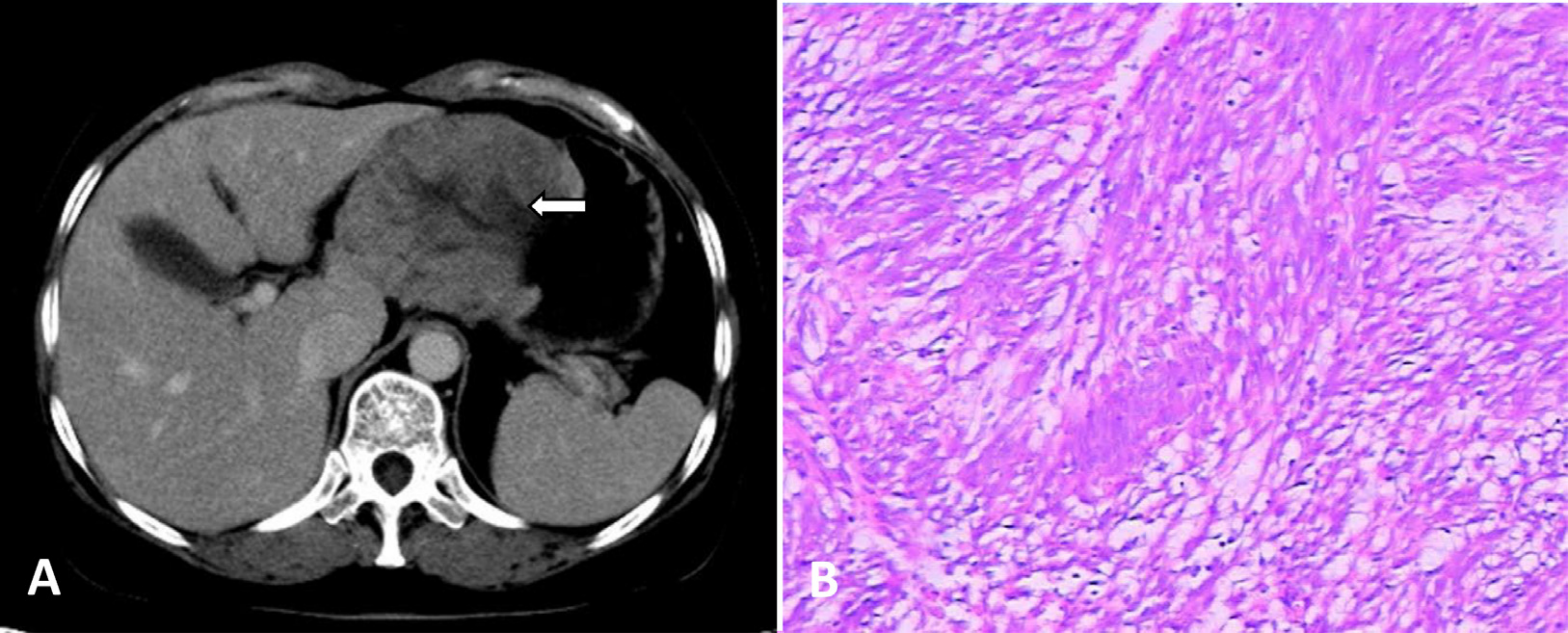
Computed tomography (CT). CT obtained on May 19, 2015, before surgery for a gastric stromal tumor revealed a gastric mass (A);Postoperative pathology revealed a high-risk “gastric” stromal tumor (B).

A 58-year-old female patient presented to the gastroenterology department of our hospital with upper abdominal pain for more than 2 months. After admission, ultrasound gastroscopy showed that the gastric body was raised and depressed, and the possibility of malignant transformation of submucosal tumors was high. Computed tomography (CT) examination revealed a dense soft tissue mass in the stomach body with uneven enhancement, which was considered a tumor (Fig. 1A). No abnormal density shadow was found under the liver capsule by CT. After preoperative evaluation, the patient underwent tumor resection. Postoperative pathology revealed a high-risk “gastric” stromal tumor (Fig. 1B). Immunohistochemistry was positive for CD117 (++), CD34 (++), DOG1 (+), vimentin (++), NSE (-), SMA (-), C-erbB-2 (-), and ki67 tumor cells <5%. A routine abdominal CT scan was performed to assess the postoperative status of her gastric stromal tumor.

**Fig. 2:**
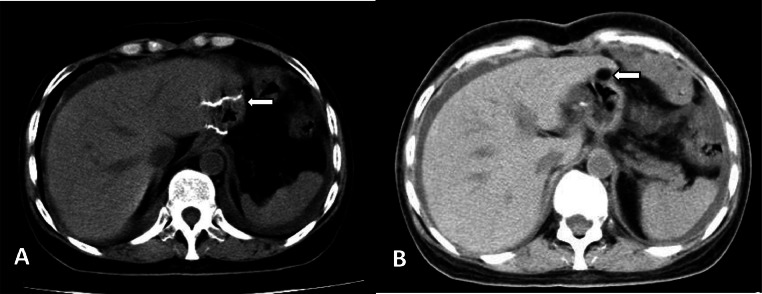
Postoperative imaging. On August 17, 2015, the first review after gastric stromal tumor surgery (A); Left liver subcapsular fat density lesions (B).

In August 2015, a lesion measuring 1.1 × 0.8 cm was identified beneath the capsule of the left lobe of the liver, exhibiting a fat density of -70 HU (Fig. 2B). The lesion was benign and may have been a lipoma or pseudolipoma.

**Fig. 3:**
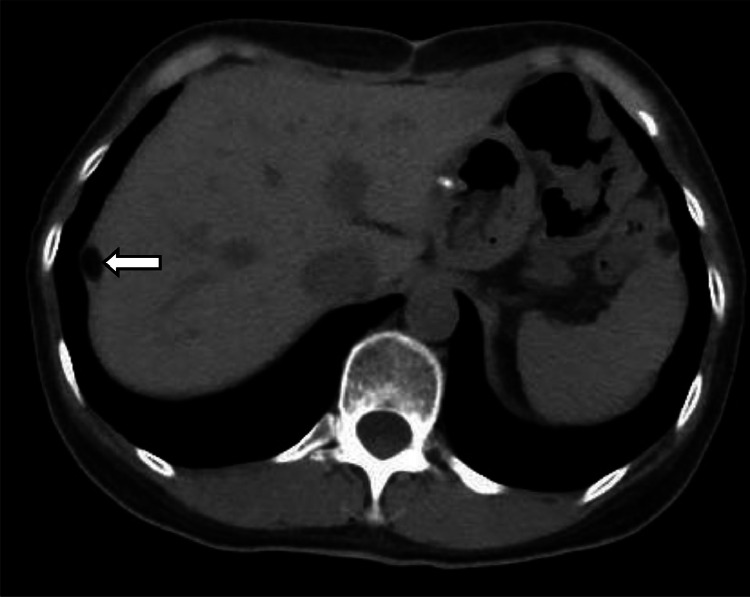
Postoperative reexamination On March 22, 2017, postoperative re-examination showed a dense, fatty lesions under the right hepatic capsule.

In March 2017, a subsequent abdominal CT scan revealed the presence of a 1.1 × 0.8 cm dense, fat-containing lesions beneath the liver capsule in the posterior upper segment of the right lobe (Fig. 3), while the previously observed lesions beneath the capsule of the left lateral lobe had resolved.

**Fig. 4:**
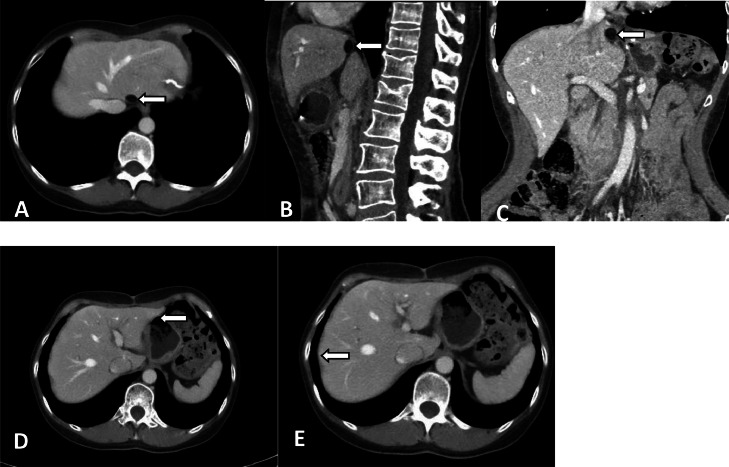
Reexamination using computed tomography (CT). On May 9, 2018, re-examination via CT showed a dense, fatty lesions (A-C) in the space between the left and caudal lobes of the liver under the hepatic capsule. The lesions in the left and right lobes of the original liver had disappeared (D, E).

In May 2018, an abdominal CT scan showed a similar dense, fatty lesions of comparable size (Figs. 4A-C) subperitoneally adjacent to the caudate lobe of the liver. The previously observed lesions in the left and right lobes of the liver had resolved (Fig. 4, Fig. 4). The internal fatty density, characteristic location within Glisson's capsule, and multiple migrations supported the diagnosis of PGC originating from Glisson's capsule.

## Discussion

The liver capsule has 2 layers, previously thought to be the outer serous layer and the inner layer called Glisson's capsule. The serous layer covers most of the liver, except the bare area on the diaphragmatic surface, porta hepatis, and the attachment site of the gallbladder. Glisson's capsule is a fibrous layer made of type III collagen that surrounds the entire liver. This anatomic feature, along with other anatomic and physiologic features of the liver, predisposes the liver capsule to a variety of pathologies [6].

**Fig. 5:**
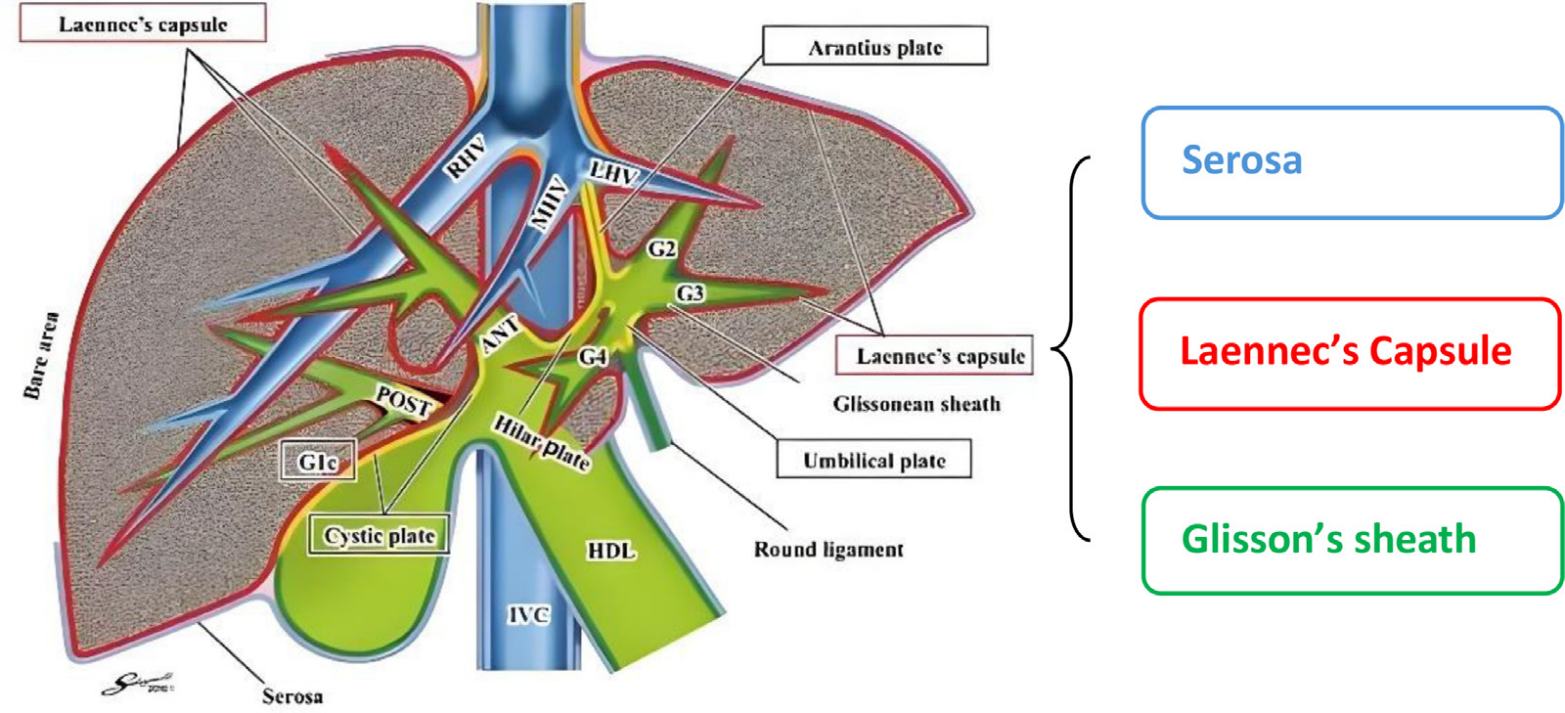
Anatomical diagram of the liver capsule.

Recent postmortem histological findings have revealed that Glisson's capsule is not a continuation of Glisson's sheath, but rather an extension of the Laennec's capsule in the porta hepatis region. The Laennec's Capsule extends into the surrounding area of Glisson's sheath [[7], [8], [9], [10], [11]]. Currently, the liver capsule is divided into an outer serous layer and an inner fibrous layer called the Laennec's capsule (Fig. 5).

PGC is an uncommon condition (0.2% prevalence) consisting of a benign hepatic lesion, usually in older males with an average age of 67 years [3,4]. PGC is thought to result from the degeneration or displacement of the omental appendage [4]. Pathological examination shows that PGC is indistinguishable from the omental appendage [4]. In CT imaging, these lesions typically manifest as well-defined fatty lesions on the liver surface with CT values ranging approximately between −10 to −100 HU [12]. CT findings of Pseudolipoma of Glisson's capsule with calcification have not been reported. However, the author occasionally found subcapsular fatty density lesions accompanied by calcification during work. However, since the movement of the lesions was not followed up nor confirmed by pathological results after surgery, whether Pseudolipoma of Glisson's capsule was accompanied by calcification needs to be further confirmed.

Patients are usually asymptomatic but may experience abdominal pain resembling appendicitis or diverticulitis [4]. Prior abdominal surgical history being a risk factor for this condition is contentious [3,4]. Interestingly, this is supported by the current case, in which this lesion was not present in the subcapsular liver before gastric tumor surgery.

Distinguishing PGC from peritoneal metastasis and solitary necrotic nodules is essential [6]. Low fat density and subcapsular migration are crucial features for differential diagnosis [13]. This case illustrates the migration of a pseudolipoma over more than 2 years across different locations on the liver surface. To the best of our knowledge, this is the second documented case of lesions migration over time. Another patient who reported PGC movement over time was an 81-year-old male patient with lung cancer. When abdominal CT examination was completed to determine whether there was abdominal metastasis, the liver subcapsular fat density lesions was found. The liver subcapsular fat attenuation lesions was found again 2 months later, and it was not in the same location as the first time [14]. Both reports support the hypothesis that isolated appendages migrate in the space between the outer serosal layer and the Glisson's capsule.

Location and internal fat density are extremely important characteristics used to narrow down differential diagnoses via CT imaging. The migratory nature of this pseudolipoma highlights another factor to consider when diagnosing subcapsular and fatty hepatic lesions. Furthermore, awareness of the possibility of migration can aid radiologists in making accurate diagnoses.

## Conclusion

PGC is a benign subcapsular hepatic lesion characterized by degenerated fat, possibly originating from displaced or degenerated liver fat tumors of the omentum. Pseudolipomas can migrate within the subcapsular region, aiding in their differentiation from other malignant lesions, such as metastases that may be located within the subcapsular area but remain fixed.

## Patient consent

This case report is in complete adherence with pertinent laws and ethical principles, while honoring the desires and privacy of the patient.

## Ethics approval

The case report was conducted in accordance with the principles of the Declaration of Helsinki and was approved by the institutional review board of [The Third People's Hospital of Zigong, Sichuan Province, China].
